# Global Prevalence of Anxiety in Gastroenterology and Hepatology Outpatients: A Systematic Review and Meta-Analysis

**DOI:** 10.1007/s11894-025-00963-x

**Published:** 2025-02-27

**Authors:** Ben Storer, Monique Holden, Kelly Ann Kershaw, Taylor A. Braund, Cassandra Chakouch, Matthew James Coleshill, Sam Haffar, Samuel Harvey, Gemma Sicouri, Jill Newby, Michael Murphy

**Affiliations:** 1 Clinical Research Department, The Black Dog Institute, Sydney, Australia; 2 School of Clinical Medicine, Faculty of Medicine and Health, UNSW, Sydney, Australia; 3 School of Psychology, Faculty of Science, UNSW, Sydney, Australia

**Keywords:** Anxiety disorders, Gastroenterology, Hepatology, Outpatients, Psychiatry

## Abstract

**Purpose of Review:**

Many patients with chronic health conditions experience anxiety, which can have significant implications on physical health outcomes and quality of life. This systematic review and meta-analysis aimed to examine the prevalence of anxiety in gastroenterology and hepatology outpatients, across factors such as physical health condition, type of anxiety, and patient demographics, with the intention to support clinicians in providing effective patient care.

**Recent Findings:**

Several recent systematic reviews have been published investigating rates of anxiety in different outpatient settings, and have found consistently high rates across the dermatology, endocrinology, cardiology and respiratory/sleep medicine fields, ranging between 25.1% and 30.3%. Whilst there are established links between gastroenterology and hepatology conditions with anxiety, there has yet to be a study estimating the overall global prevalence of anxiety in this outpatient setting.

**Summary:**

PubMed, Embase, Cochrane and PsycINFO databases were searched from database inception to January 2023 for studies reporting anxiety in gastroenterology and hepatology outpatients ≥ 16 years of age. Prevalence was extracted from self-report questionnaires, diagnostic interviews, and records. The final meta-analysis included 81 studies, with 28,334 participants. Pooled prevalence of anxiety was 31.2% (95% CI 28.2%—34.4%). Subgroup analyses identified significant differences in prevalence across anxiety type, with health anxiety showing the highest prevalence at 23.7%, followed by generalised anxiety 14.5%, specific phobia 12.5%, panic disorder/agoraphobia 12.2%, social anxiety 11.3%, post-traumatic stress disorder 4.9%, and obsessive-compulsive disorder 4.2%. No other significant differences were found. Anxiety is thus common amongst gastroenterology and hepatology outpatients, and so it is important that careful consideration be given to the identification and management of anxiety in these settings.

**Supplementary Information:**

The online version contains supplementary material available at 10.1007/s11894-025-00963-x.

## Introduction

Anxiety disorders are some of the most common mental disorders worldwide [1], with a recent study estimating the global prevalence to be approximately 4.8% [2]. This tends to vary across a range of demographic variables, however, for example, one’s age, sex, socioeconomic status and residing country can influence prevalence rates [2–4]. Evidence indicates that anxiety disorders have a significant impact on an individual’s quality of life and functioning [5, 6], and they have been found to have an economic burden to society as a whole [7]. Therefore, it remains crucial to understand which population groups may be particularly vulnerable to anxiety disorders, to ensure appropriate screening, assessment, and treatment.

Several studies have pointed to a higher anxiety prevalence estimate for those with physical health conditions [4]. It is often proposed that the relationship between the two is bidirectional [8–10], with both physical health conditions leading to anxiety, and anxiety also exacerbating medical symptoms and conditions. Although the precise mechanism is not known, physical health conditions and anxiety are thought to be interrelated through a complex interaction of biological (e.g., chronic inflammation, neuroendocrine dysregulation), psychological (e.g., negative and catastrophic thinking patterns and avoidance behaviours) and social mechanisms (e.g., stigma, isolation, loneliness and financial stress) [11, 12]. A growing body of research into the gut-brain axis also suggests that there are complex bidirectional communication networks between the gastrointestinal tract and brain that may heighten the prevalence of anxiety and stress in patients with gastroenterology and hepatology disorders [13, 14]. However, many studies investigating the prevalence of anxiety in the medically unwell [4, 15, 16] have tended to combine patients from different medical settings, for example, those that are inpatients, outpatients, and in primary care. This reduces the specificity of the results, especially given the variation in experiences and stressors associated with each setting [17]. Several recent systematic reviews have been published seeking to address this in the outpatient setting, and have found anxiety rates of 26.7% for dermatology [18], 25.1% for endocrinology [19], 28.9% for cardiology [20], and 30.3% for respiratory and sleep conditions [21].

Importantly, a growing body of work has also highlighted anxiety as common in those experiencing gastroenterology and hepatology disorders. In a United Kingdom (UK) based study, the prevalence rate of anxiety for those falling under this speciality was found to be approximately 19.5% [22]. However, this study was limited to the UK context, combined different medical clinics, and only investigated rates of health anxiety (i.e., illness anxiety disorder and somatic symptom disorder). There has yet to be a study estimating the overall *global* prevalence of anxiety in the gastroenterology and hepatology outpatient setting. Having knowledge of this would be helpful in several ways – helping guide clinical practice, and promoting more personalised and comprehensive treatments, which could improve patient satisfaction with care [23].

Studies looking at particular gastroenterological disorders, including upper gastrointestinal (GI) disorders such as gastroesophageal reflux disease, lower GI disorders such as inflammatory bowel disease (IBD), and disorders of the gut-brain interaction (DGBI), which includes Irritable Bowel Syndrome (IBS), as per the ROME IV criteria [24, 25], have been conducted. The few systematic reviews and meta-analyses looking at these disorders have suggested that rates of anxiety sit at around 39.1% for IBS [26, 27], 34.4% for gastroesophageal reflux diseases [28], and between 32.1% and 35.1% for IBD [29, 30]. There are mixed findings relating to the differences between Ulcerative Colitis and Crohn’s Disease that fall under IBD, however, with some research finding similar estimates [29], and others finding significant, yet only modestly higher rates for those with Crohn’s Disease [31]. Importantly, a recent consensus statement within the context of IBD highlighted that crucial need for mental health care and support in this setting [32].

Rates of anxiety similarly appear to be elevated for patients within the hepatology setting. For example, one study noted that the prevalence of anxiety for those with hepatitis C was approximately 24% [33], with another finding even higher rates for those with chronic hepatitis C at 41% [34]. In a study based in the United States, 42.6% of patients with liver cirrhosis were found to have moderate-to-severe anxiety levels [35] and those with chronic pancreatitis have been shown to have a higher odds of developing anxiety than those without [36]. These studies, however, have similar limitations to the gastroenterology literature, and there is no past comprehensive review study estimating the prevalence of anxiety within this speciality overall.

The current study thus aims to provide insight into the prevalence of anxiety in gastroenterology and hepatology outpatient settings across the literature. It seeks to examine trends in the prevalence across different health conditions, types of anxiety, and relevant demographic and socioeconomic variables. In doing so, it aims to support health professionals in providing optimal care by understanding trends amongst patients in their clinic, in the hopes to improve patient wellbeing and quality of life.

## Methods

### Design and Registration

This project was conducted in accordance with the Preferred Reporting Items for Systematic Reviews and Meta-Analyses (PRISMA) 2020 Statement [37]. It was registered in the International Prospective Register of Systematic Reviews (PROSPERO; CRD42021282416).

### Search Strategy and Eligibility Criteria

**Fig. 1:**
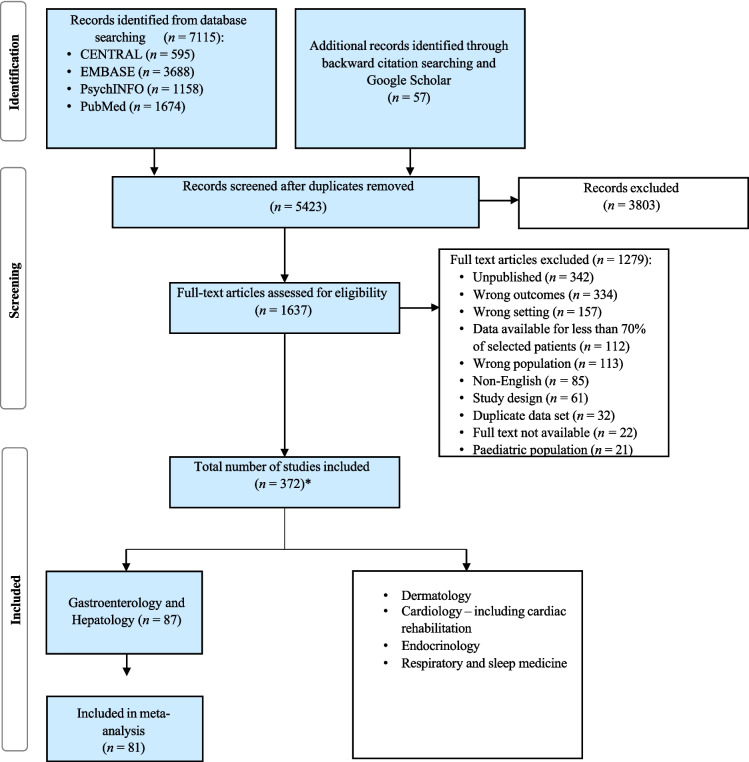
PRISMA flowchart

PubMed, PsycINFO, Cochrane Library and EMBASE were searched. The final search date was the 23rd of January 2023. Search strategy was developed with UNSW Sydney librarians, using relevant clinical, study type, anxiety and outpatient keywords (Appendix A). This was part of a review of the prevalence of anxiety across five medical outpatient settings: dermatology, cardiology, gastroenterology and hepatology, endocrinology, and respiratory and sleep medicine. Given the large volume of studies (Fig. 1), each specialty was reviewed separately for more thorough analysis.

To be eligible for inclusion, studies had to report data on medical outpatients ≥ 16 years of age. This data had to include prevalence of anxiety symptoms and/or disorders, assessed by either a validated self-report tool, diagnostic interview/criteria, or clinician report. Primary care, inpatient, emergency, surgical, palliative, end-of-life, oncology, and fertility clinics/settings were excluded. Studies that primarily focussed on psychiatric, addiction and other mental health patients were excluded. Where full-text papers were unavailable, authors were contacted, and if no response was received in two weeks, the study was excluded.

### Study Selection and Data Extraction

Covidence web-based systematic review software [38] was used for screening and extraction. Screening, both at the level of title and abstract, and then full text review, was completed by two independent researchers (any two of BS, CC, KAK, GS, MJC, SH, TB). Extracted data included information about design, country, demographics, medical condition/s, method of identification of anxiety, outcome measures (and cut offs if reported), anxiety types, and prevalence. Extraction and quality assessment were conducted by two reviewers (any two of MH, KAK, BS), and conflicts resolved by a third reviewer (MM, BS).

### Quality Assessment

The Joanna Briggs Institute (JBI) critical appraisal tool for prevalence studies [39] was used to assess the methodological quality and determine any risk of bias. The tool consists of nine questions, with a scoring key of “yes”, “no”, “unclear”, or “not applicable”. The quality of the literature was divided as follows: high (> 5 “yes” scores), moderate (3–5 “yes” scores), and low (0–2 “yes” scores). Higher scores indicate greater quality, and thus lower risk of bias.

### Data Synthesis and Meta-Analysis

Meta-analysis was performed in Comprehensive Meta Analysis Version 4 (CMA 4) software [40]. A random-effects model was used to pool prevalence estimates, and a mixed-effects model was applied to preplanned subgroup analyses. This included relationship of prevalence with condition, anxiety type, method of identification, country, sex, publication year, and age. Results are reported as effect size estimates with 95% confidence intervals (CIs). The proportion of variance was assessed using the *I*^*2*^ statistic, and funnel plots and the Egger’s regression intercept [41] were reviewed to assess publication bias. Relative weights were calculated based on the inverse of overall study error variance.

## Results

### Study Characteristics

The initial search identified 5440 studies (Fig. 1), with 81 meeting inclusion criteria for meta-analysis. The 81 included studies were published 1996–2022, with total *n* = 28,334 participants. 20 studies scored the maximum 9 on the JBI quality assessment, 30 scored 8, 23 scored 7, 7 scored 6 and 1 scored 5, meaning that all 81 studies achieved the JBI quality assessment requirements for inclusion. Study types included cross-sectional (*n* = 61), cohort (*n* = 7), non-randomised interventional (*n* = 5), case control (*n* = 4), prevalence (*n* = 2), psychometric (*n* = 1), and randomised controlled trial (*n* = 1). Sample sizes ranged from *n* = 21 to *n* = 8,924, with a mean age of 44.48 (*SD* 11.66), and 54.37% female participants. Most studies were conducted in Europe (*n* = 30), followed by North America (*n* = 22), Asia (*n* = 9), Oceania (*n* = 7), Middle East (*n* = 7), and South America (*n* = 5). One multicentre study combined data across two different countries. 64 studies were conducted in “developed” countries according to the United Nations Human Development Index (HDI) [42], while 17 were considered to be “developing”.

Methods of identification for anxiety included self-report measures (*n* = 68), diagnostic interviews (*n* = 9), patient notes (*n* = 3), and unstructured interview (*n* = 1). Most investigated prevalence of anxiety symptoms or disorders overall (*n* = 73), however, some reported on anxiety types, including generalised anxiety (*n* = 14), health anxiety (including illness anxiety disorder/somatic symptom disorder/hypochondriasis/somatisation) (*n* = 12), panic and agoraphobia (*n* = 10), specific phobias (*n* = 6), social anxiety (*n* = 5), obsessive compulsive disorder (*n* = 4), and post-traumatic stress (*n* = 4). Studies were conducted for a range of specific conditions, including inflammatory bowel disease (IBD) (*n* = 29), disorders of gut-brain interaction (DGBI) including IBS (*n* = 23), hepatitis (*n* = 10), and reflux conditions (*n* = 5). 13 studies investigated the prevalence within general gastroenterology and hepatology patients. Study characteristics are summarised in Table 1.

**Table 1:** Characteristics of studies included in main meta-analysis

FIRST AUTHOR (YEAR)	COUNTRY	STUDY/PERIOD	MEAN AGE (SD); SEX % FEMALE	CONDITION	OUTCOME MEASURE/CUT OFF	METHOD OF INDENTIFICATION	ANXIETY TYPE	ANXIETY PREVALENCE % (N/N)
ABU SNEINEH (2022)	Jordan	Cross Sectional 2017–2018	39.30 (13.64); 68.60%	IBD	GAD7 ≥ 5	Self-Report	Generalised Anxiety Symptoms/Disorder	65.71% (46/70)
ALOSAIMI (2014)	Saudi Arabia	Cross Sectional 2013	35.80 (12.60); 31.00%	General	GAD7 ≥ 10	Self-Report	Generalised Anxiety Symptoms/Disorder	50.23% (220/438)
BANOVIC (2012)	France	Cross Sectional	35.06 (11.74); 47.00%	IBD	HADS ≥ 8	Self-Report	Anxiety Symptoms/Disorder	24.69% (20/81)
BAYRAK (2020)	Turkey	Case Control 2019	37.90 (12.10); 49.40%	DGBI	BAI ≥ 8	Self-Report	Anxiety Symptoms/Disorder	39.67% (123/310)
BENNEBROEK EVERTSZ’ (2012)	Netherlands	Cross Sectional 2006–2008	43.40 (13.80); 56.30%	IBD	HADS ≥ 8	Self-Report	Anxiety Symptoms/Disorder	35.06% (81/231)
BERENS (2020)	Germany	Cross Sectional 2012–2015	NR (NR); 72.00%	DGBI	GAD7 NR	Self-Report	Anxiety Symptoms/Disorder	17.71% (48/271)
BJORKMAN (2015)	Sweden	Cross Sectional 2002–2010	40.30 (NR); 72.70%	DGBI	HADS ≥ 11	Self-Report	Anxiety Symptoms/Disorder	21.01% (117/557)
BLEWETT (1996)	United Kingdom	Cross Sectional	NR (NR): 68.30%	DGBI	HADS ≥ 11	Self-Report	Anxiety Symptoms/Disorder	66.67% (42/63)
BOGALE (2022)	United States	Case Control 2018–2020	39.00 (10.00); 63.90%	IBD	HADS ≥ 8	Self-Report	Anxiety Symptoms/Disorder	55.56% (20/36)
BRANDI (2009)	Brazil Brazil	Cross Sectional 2005–2007 Cross Sectional 2005–2007	38.20 (10.8); 50.00% 39.90 (13.90); 53.00%	IBD Reflux Condition/s	HADS ≥ 8 HADS ≥ 8	Self-Report Self-Report	Anxiety Symptoms/Disorder Anxiety Symptoms/Disorder	33.64% (37/110) 22.73% (25/110)
BRAY (2019)	Australia	Non-Randomised Interventional	39.60 (NR); 83.00%	DGBI	HADS ≥ 8	Self-Report	Anxiety Symptoms/Disorder	73.33% (22/30)
BRAY (2022)	Australia	Cohort 2017–2019	42.00 (14.77); 81.00%	DGBI	HADS ≥ 8	Self-Report	Anxiety Symptoms/Disorder	70.45% (31/44)
BYRNE (2017)	Canada	Cross Sectional 2016	38.71 (14.46); 50.60%	IBD	GAD7 ≥ 10	Self-Report	Generalised Anxiety Symptoms/Disorder	21.12% (68/322)
CALIXTO (2018)	Brazil	Cross Sectional	41.20 (13.00); 58.30%	IBD	HADS NR	Self-Report	Anxiety Symptoms/Disorder	55.66% (59/106)
CALVO (2021)	Spain	Cross Sectional	43.70 (12.90); 45.10%	IBD	HADS ≥ 8	Self-Report	Anxiety Symptoms/Disorder	34.31% (35/102)
CHAN (2017)	Singapore	Cross Sectional 2014–2015	43.80 (15.40); 31.00%	IBD	HADS ≥ 8	Self-Report	Anxiety Symptoms/Disorder	24.00% (48/200)
CURY (2022)	Brazil	Cross Sectional 2018–2019	33.71 (10.08); 63.00%	IBD	HADS ≥ 9	Self-Report	Anxiety Symptoms/Disorder	40.00% (40/100)
DE BOER (2016)	Netherlands	Cross Sectional	41.00 (12.00); 57.00%	IBD	HADS ≥ 8	Self-Report	Anxiety Symptoms/Disorder	36.14% (73/202)
ELMOEZ (2014)	Egypt	Cross Sectional	53.10 (5.90); 75.00%	Hepatitis	SCL-12-R NR	Self-Report	Health Anxiety Symptoms/Disorder	33.33% (16/48)
FARROKHYAR (2006)	Canada	Cross Sectional	NR (NR); 54.40%	IBD	HADS ≥ 7	Self-Report	Anxiety Symptoms/Disorder	26.85% (40/149)
FORD (2014)	Canada	Cross Sectional 2008–2012	NR (NR); 67.90%	DGBI	HADS ≥ 11	Self-Report	Anxiety Symptoms/Disorder	25.18% (250/993)
FRANCO (2022)	Brazil	Cross Sectional 2017–2018	50.41 (12.94); 50.00%	IBD	HADS ≥ 8	Self-Report	Anxiety Symptoms/Disorder	52.22% (47/90)
FREITAS (2015)	Brazil	Cross Sectional	45.10 (14.08); 57.10%	IBD	HADS ≥ 8	Self-Report	Anxiety Symptoms/Disorder	25.17% (37/147)
FRESAN ORELLANA (2021)	Mexico	Cross Sectional 2019	45.00 (12.10); 57.50%	IBD	HADS ≥ 8	Self-Report	Anxiety Symptoms/Disorder	28.00% (56/200)
GARCIA-ALANIS (2021)	Mexico	Cross Sectional	NR (NR); 48.10%	IBD	SCID	Diagnostic Interview	Anxiety Symptoms/Disorder	44.23% (46/104)
GOODOORY (2022)	United Kingdom	Cohort 2016–2020	34.80 (14.00); 76.80%	DGBI	HADS ≥ 11	Self-Report	Anxiety Symptoms/Disorder	49.33% (185/375)
GUZ (2008)	Turkey	Cross Sectional 2002	NR (NR); 54.50%	General	SCID	Diagnostic Interview	Anxiety Symptoms/Disorder	10.00% (9/90)
HADI (2021)	United States	Cohort 2009–2018	40.68 (12.08); 77.38%	DGBI	-	Patient Notes	Anxiety Symptoms/Disorder	36.65% (81/221)
HANSON (2009)	United States	Case Control 1999–2002	NR (NR); 54.50%	IBD	Other	Self-Report	Anxiety Symptoms/Disorder	13.00% (26/200)
HILSABECK (2003)	United States	Cross Sectional	46.30 (6.60); 42.90%	Hepatitis	BAI ≥ 8	Self-Report	Anxiety Symptoms/Disorder	47.62% (10/21)
HUANG (2018)	China	Cross Sectional 2016–2017	58.50 (11.40); 31.80%	Liver Cirrhosis	HADS ≥ 8	Self-Report	Anxiety Symptoms/Disorder	11.49% (17/148)
JACKSON (2018)	Australia	Cross Sectional 2015	34.00 (10.90); 49.00%	IBD	HADS ≥ 8	Self-Report	Anxiety Symptoms/Disorder	45.68% (37/81)
KALAITZAKIS (2008)	Sweden Sweden	Cross Sectional Cross Sectional	NR (NR); NR NR (NR); NR	Short Bowel Syndrome IBD	HADS ≥ 8 HADS ≥ 8	Self-Report Self-Report	Anxiety Symptoms/Disorder Anxiety Symptoms/Disorder	7.69% (2/26) 7.32% (3/41)
KANURI (2016)	United States United States	Cross Sectional 2009–2013 Cross Sectional 2009–2013	47.60 (0.90); 81.00% 51.60 (1.00); 65.00%	DGBI Non-DGBI	BAI ≥ 16 BAI ≥ 16	Self-Report Self-Report	Anxiety Symptoms/Disorder Anxiety Symptoms/Disorder	31.25% (85/272) 13.82% (34/246)
KARPIN (2021)	United States	Cross Sectional	NR (NR); 55.90%	IBD	CAT-MH ≥ 35	Self-Report	Anxiety Symptoms/Disorder	13.43% (18/134)
KAWOOS (2017)	India	Case Control 2014–2015	39.70 (11.40); 72.50%	DGBI	MINI	Diagnostic Interview	Anxiety Symptoms/Disorder	10.63% (17/160)
KEEFER (2008)	United States	Cross Sectional	45.90 (15.50); 72.00%	General	HADS ≥ 8	Self-Report	Anxiety Symptoms/Disorder	40.25% (64/159)
KESEN (2019)	Turkey	Non-Randomised Interventional	58.90 (10.51); 61.60%	Hepatitis	HADS ≥ 10	Self-Report	Anxiety Symptoms/Disorder	24.66% (18/73)
KESSING (2015)	Netherlands	Cohort 2011–2012	58.00 (NR); 63.10%	Reflux Condition/s	HADS ≥ 8	Self-Report	Anxiety Symptoms/Disorder	31.56% (71/225)
KNOWLES (2011)	Australia	Cross Sectional	37.80 (13.70); 64.60%	IBD	HADS ≥ 9	Self-Report	Anxiety Symptoms/Disorder	65.63% (63/96)
KRUIMEL (2015)	Netherlands	Cohort 2009–2011	48.00 (17.10); 70.20%	DGBI	-	Patient Notes	Anxiety Symptoms/Disorder	49.19% (61/124)
KUMAR (2021)	India	Cross Sectional 2015–2017	48.30 (10.50); 8.00%	Liver Cirrhosis	GAD7 ≥ 8	Self-Report	Generalised Anxiety Symptoms/Disorder	34.06% (374/1098)
LUO (2017)	China	Cross Sectional 2014–2015	34.10 (11.20); 37.00%	IBD	HADS ≥ 8	Self-Report	Anxiety Symptoms/Disorder	24.66% (54/219)
MACLEAN (2012)	United States	Psychometric	34.60 (11.70); 82.00%	DGBI	CMCQ	Self-Report	Anxiety Symptoms/Disorder	17.13% (49/286)
MANCINI (2021)	Italy	Cross Sectional 2015–2016	60.80 (9.40); 50.00%	General	HADS ≥ 8	Self-Report	Anxiety Symptoms/Disorder	40.82% (20/49)
MANSUETO (2021)	Italy	Cross Sectional 2019	NR (NR); 46.01%	General	Other	Self-Report	Anxiety Symptoms/Disorder	14.11% (69/489)
MARAFINI (2020)	Italy	Cross Sectional 2018–2019	NR (NR); 46.00%	IBD	MINI	Diagnostic Interview	Anxiety Symptoms/Disorder	11.39% (27/237)
MARINELLI (2020)	Italy	Cross Sectional 2018–2019	44.30 (13.23); 47.30%	IBD	HADS ≥ 8	Self-Report	Anxiety Symptoms/Disorder	41.89% (31/74)
MIKOCKA-WALUS (2008)	Australia	Cross Sectional 2005–2006	54.35 (13.83); 78.00%	DGBI	HADS ≥ 8	Self-Report	Anxiety Symptoms/Disorder	50.00% (16/32)
MULDER (2000)	New Zealand	Non-Randomised Interventional	34.23 (7.20); 28.57%	Hepatitis	SCID	Diagnostic Interview	Panic/Agoraphobia Symptoms/Disorder	26.98% (17/63)
PINTO-SANCHEZ (2015)	Canada	Cross Sectional 2008–2012	48.27 (17.22); 62.50%	DGBI	HADS ≥ 11	Self-Report	Anxiety Symptoms/Disorder	27.46% (659/2400)
POLSTER (2017)	Sweden	Cross Sectional	35.00 (NR); 71.50%	DGBI	HADS ≥ 11	Self-Report	Anxiety Symptoms/Disorder	34.18% (54/158)
PONTONE (2015)	Italy	Cross Sectional 2009–2010	53.30 (17.00); 60.70%	General	STAI	Self-Report	Anxiety Symptoms/Disorder	25.41% (31/122)
PORCELLI (1998)	Italy	Cross Sectional	38.80 (15.80); 65.40%	DGBI	HADS ≥ 11	Self-Report	Anxiety Symptoms/Disorder	48.03% (61/127)
PORCELLI (2000)	Italy	Cross Sectional	37.50 (13.80); 64.20%	DGBI	SCID	Diagnostic Interview	Anxiety Symptoms/Disorder	14.74% (28/190)
PORCELLI (2010)	Italy	Cross Sectional	36.10 (9.40); 44.00%	IBD	HADS ≥ 8	Self-Report	Anxiety Symptoms/Disorder	33.70% (31/92)
PRADEEP (2020)	India	Non-Randomised Interventional 2016–2017	NR (NR); 48.00%	Reflux Condition/s	1CD-10	Diagnostic Interview	Anxiety Symptoms/Disorder	81.25% (65/80)
RENGARAJAN (2021)	France; United States	Cohort 2015–2017	NR (NR); 70.48%	Reflux Condition/s	HADS NR	Self-Report	Anxiety Symptoms/Disorder	53.33% (56/105)
RIOLI (2019)	Italy	Cross Sectional 2015–2016	61.30 (9.20); 50.00%	General	HADS ≥ 8	Self-Report	Anxiety Symptoms/Disorder	30.19% (16/53)
SAYUK (2007)	United States United States	Cross Sectional 2003–2005 Cross Sectional 2003–2005	45.60 (1.20); 70.10% 45.30 (1.60); 47.90%	DGBI General	- -	Unstructured Interview Unstructured Interview	Anxiety Symptoms/Disorder Anxiety Symptoms/Disorder	6.42% (12/187) 7.14% (10/140)
SCHAFER (2005)	Germany	Cross Sectional	42.50 (11.20); 49.50%	Hepatitis	HADS ≥ 11	Self-Report	Anxiety Symptoms/Disorder	13.59% (14/103)
SCHRAMM (2014)	Germany	Cross Sectional 2008–2009	50.00 (NR); 71.00%	Hepatitis	GAD7 ≥ 10	Self-Report	Generalised Anxiety Symptoms/Disorder	12.62% (13/103)
SINGH (2019)	United States	Cross Sectional 2017–2018	43.00 (17.10); 80.30%	DGBI	PROMIS ≥ 60	Self-Report	Anxiety Symptoms/Disorder	35.77% (49/137)
SIUPSINSKIENE (2007)	United States	Non-Randomised Interventional	NR (NR); 75.00%	Reflux Condition/s	HADS ≥ 11	Self-Report	Anxiety Symptoms/Disorder	30.00% (30/100)
STEWART (2012)	Australia	Cross Sectional 2006–2010	45.20 (11.00); 27.80%	Hepatitis	HADS ≥ 8	Self-Report	Anxiety Symptoms/Disorder	63.54% (251/395)
STIGLIANO (2016)	Italy	Cross Sectional	65.00 (13.00); 48.00%	Pancreatic Disorders	NR	Self-Report	Anxiety Symptoms/Disorder	36.11% (39/108)
TUNCALI (2018)	Turkey	Cross Sectional 2013–2015	60.60 (10.40); 50.00%	General	BAI ≥ 16	Self-Report	Anxiety Symptoms/Disorder	31.58% (18/57)
TUNG (2009)	China	Cross Sectional 2008	46.80 (12.00); 50.50%	DGBI	SCID	Diagnostic Interview	Anxiety Symptoms/Disorder	21.21% (21/99)
TYRER (2011)	United Kingdom	Prevalence 2008	NR (NR); NR	General	HAI ≥ 20	Self-Report	Health Anxiety Symptoms/Disorder	19.46% (1737/8924)
TYRER (2019)	United Kingdom	Prevalence 2006–2008	NR (NR); NR	General	SHAI ≥ 20	Self-Report	Health Anxiety Symptoms/Disorder	17.24% (15/87)
UDINA (2016)	Spain	Cohort 2005–2009	44.00 (10.40); 32.80%	Hepatitis	SCID	Diagnostic Interview	Anxiety Symptoms/Disorder	26.16% (90/344)
VIGANO (2016)	Italy	Cross Sectional	44.39 (11.30); 39.80%	IBD	HADS ≥ 11	Self-Report	Anxiety Symptoms/Disorder	35.77% (44/123)
VIGANO (2018)	Italy	Cross Sectional 2016–2017	47.10 (12.03); 44.70%	IBD	HADS ≥ 8	Self-Report	Anxiety Symptoms/Disorder	42.35% (72/170)
VU (2014)	United States	Cross Sectional 2006–2013	45.40 (2.10); 79.90%	DGBI	BAI ≥ 16	Self-Report	Anxiety Symptoms/Disorder	31.19% (189/606)
WABICH (2020)	United States	Cross Sectional	42.00 (NR); 63.00%	IBD	BSI ≥ 63 (T-score)	Self-Report	Anxiety Symptoms/Disorder	6.42% (7/109)
WILPART (2017)	Sweden	Cross Sectional 2003–2007	40.40 (NR); 69.90%	DGBI	HADS ≥ 11	Self-Report	Anxiety Symptoms/Disorder	19.91% (43/216)
YONGWEN NG (2018)	Canada	Cross Sectional 2010–2012	NR (NR); 64.50%	IBD	HADS ≥ 11	Self-Report	Anxiety Symptoms/Disorder	20.53% (70/341)
YOUNOSSI (2016)	United States	Non-Randomised Interventional 2014	NR (NR); 30.00%	Hepatitis	-	Patient Notes	Anxiety Symptoms/Disorder	21.72% (58/267)
ZHANG (2016)	China	Cross Sectional	NR (NR); 46.03%	General	HADS ≥ 9	Self-Report	Anxiety Symptoms/Disorder	41.45% (361/871)
ZHENG (2015)	China	Cross Sectional	NR (NR); 44.70%	General	HADS ≥ 8	Self-Report	Anxiety Symptoms/Disorder	21.54% (196/910)
ZICKMUND (2003)	United States	Cross Sectional 1998–2001	45.00 (NR); 37.00%	Hepatitis	HADS NR	Self-Report	Anxiety Symptoms/Disorder	71.21% (183/257)

### Prevalence of Anxiety Overall

**Fig. 2:**
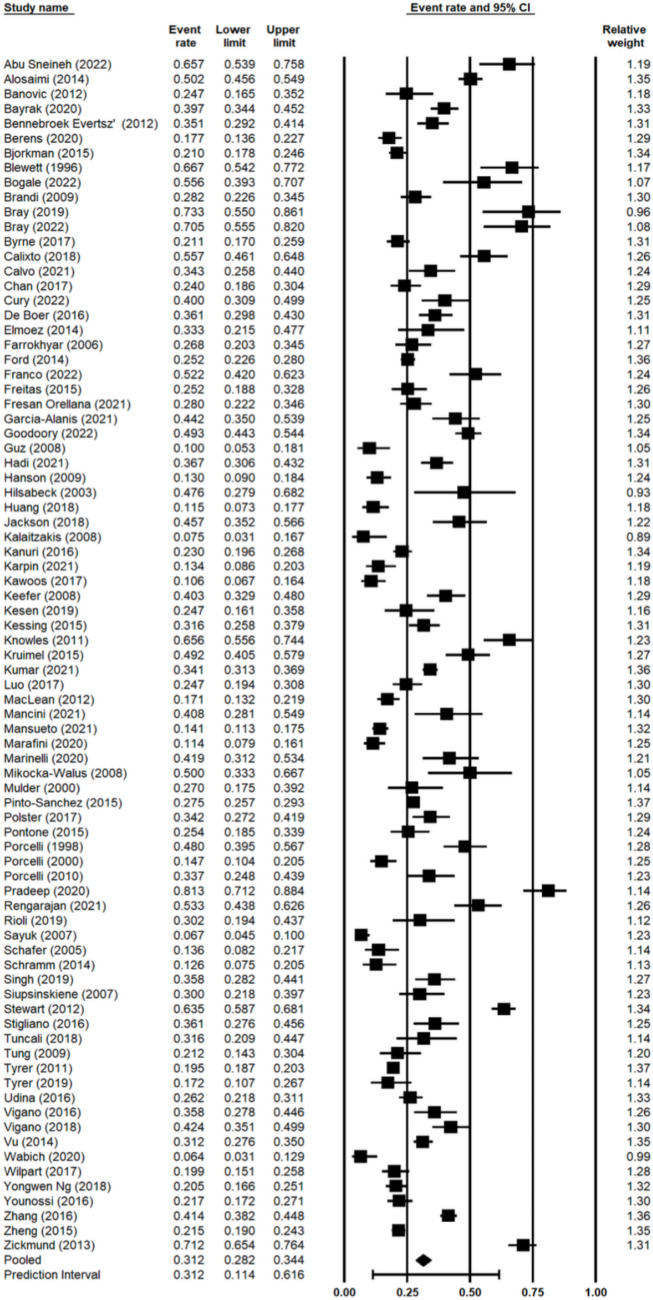
Pooled prevalence of anxiety across all studies

Under the random-effects model, the mean prevalence estimate of anxiety reported by the 81 studies was 31.2% (95% CI 28.2%—34.4%, 95% PI (prediction interval) 11.4%—61.6%) (Fig. 2). Significant between-study heterogeneity was found (*p* < 0.001,* I*^*2*^ = 95.86%). Asymmetry in the funnel plot and Egger’s test (*p* < 0.01) indicated publication bias was present in the meta-analysis (Appendix B). Using the Trim and Fill method to the left of the mean, the point estimate and 95% CI remained unchanged, however, to the right of the mean, the point estimate and 95% CI changed to 33.7% and 30.4%—37.0%. This suggested that six studies were missing to the right of the mean.

### Prevalence of Anxiety by Gastroenterology or Hepatology Condition

**Fig. 3:**
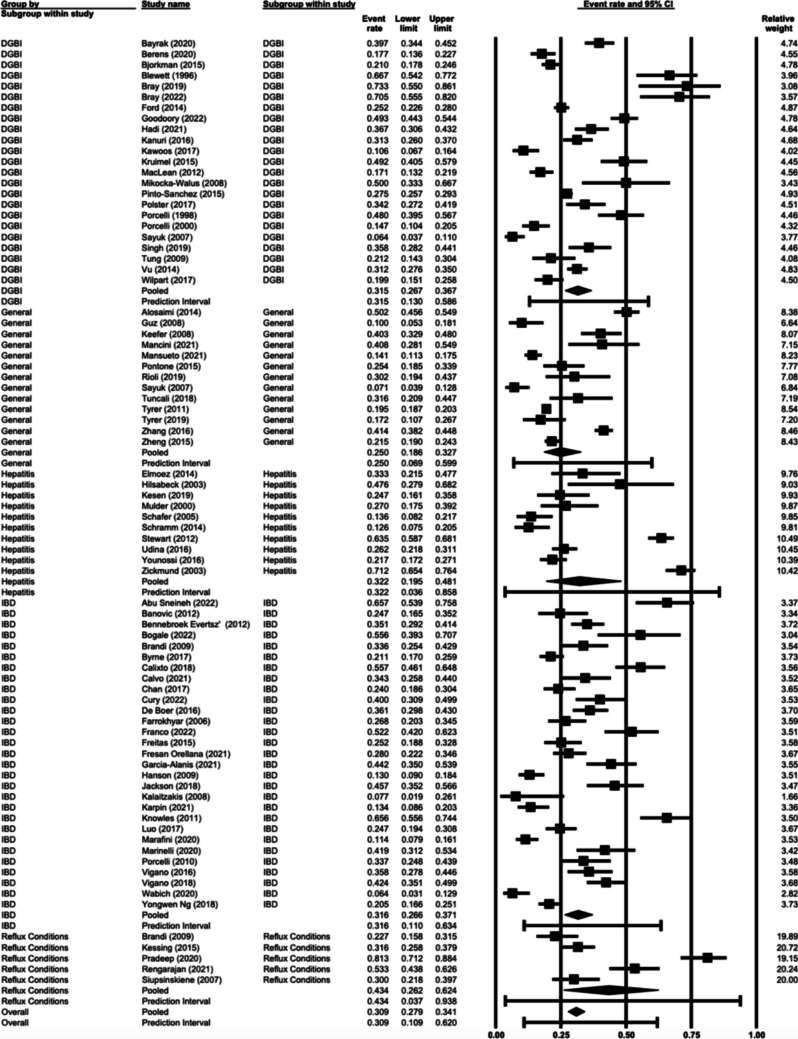
Prevalence of anxiety by gastroenterology or hepatology condition

Prevalence estimates did not vary significantly (*p* = 0.35) between different gastroenterology and hepatology conditions in the outpatient setting. Specific estimates were 43.4% (95% CI 26.2%—62.4%, *n* = 5) for reflux conditions, followed by hepatitis at 32.2% (95% CI 19.5%—48.1%, *n* = 10), IBD at 31.6% (95% CI 26.6%—37.1%, *n* = 29), DGBI at 31.5% (95% CI 26.7%—36.7%, *n* = 23) and general gastroenterology and hepatology clinic at 25.0% (95% CI 18.6%—32.7%, *n* = 13) (Fig. 3). The IBD conditions Ulcerative Colitis and Crohn’s Disease were additionally compared to one another. No significant difference (*p* = 0.47) was found between Ulcerative Colitis 31.1% (95% CI 21.0%—43.4%, *n* = 11) and those with Crohn’s Disease 36.6% (95% CI 27.6%—46.7%, *n* = 12).

### Prevalence of Anxiety by Method of Identification

No significant difference (*p* = 0.22) was found in the prevalence of anxiety when comparing studies that used self-report measures 32.6% (95% CI 29.3%—36.1%, *n* = 68) to diagnostic interviews 24.3% (95% CI 14.5%—37.7%, *n* = 9). Common self-report measures included the Hospital Anxiety and Depression Scale (HADS), General Anxiety Disorder-7 (GAD-7), and Beck Anxiety Inventory and State-Trait Anxiety Inventory. The HADS produced the highest prevalence estimate of anxiety at 36.4% (95% CI 32.2%—40.7%, *n* = 46), followed by the GAD-7 at 31.2% (95% CI 20.6%—44.3%, *n* = 6), and “others” at 23.4% (95% CI 19.3%—28.0%, *n* = 16). The difference between these groups was statistically significant (*p* < 0.001)*.* The HADS has two common cut-off scores used in the literature, 8 to (possible anxiety disorder), and 11 (likely anxiety disorder) [43]. No significant difference (*p* = 0.30) was found in studies using a threshold of 8 at 35.7% (95% CI 29.6%—42.2%, *n* = 25), and a threshold of 11 at 31.0% (95% CI 25.4%—37.4%, *n* = 12).

### Prevalence of Anxiety by Type of Anxiety

**Fig. 4:**
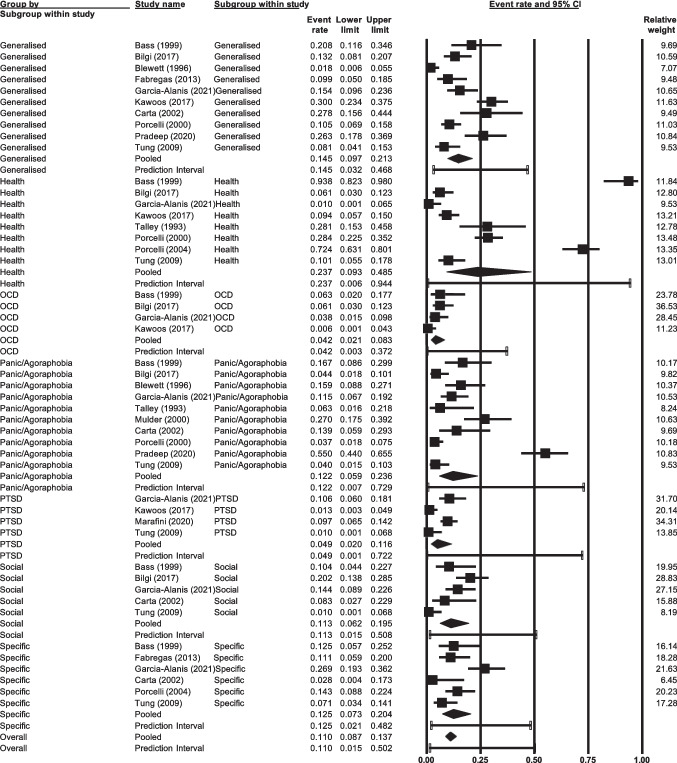
Prevalence of anxiety by type of anxiety

A significant difference (*p* < 0.05) was found in the prevalence of the different types of anxiety disorders, with the highest prevalence being health anxiety 23.7% (95% CI 9.3%—48.5%, *n* = 8), followed by generalised anxiety 14.5% (95% CI 9.7%—21.3%, *n* = 10), specific phobia 12.5% (95% CI 7.3%—20.4%, *n* = 6), panic disorder and agoraphobia 12.2% (95% CI 5.9%—23.6%, *n* = 10), social anxiety 11.3% (95% CI 6.2%—19.5%, *n* = 5), post-traumatic stress disorder 4.9% (95% CI 2.0%—11.6%, *n* = 4), and obsessive compulsive disorder 4.2% (95% CI 2.1%—8.3%, *n* = 4; Fig. 4). The majority of studies assessing for these specific anxiety types used diagnostic interviews as their method of identification, including the Structured Clinical Interview for the DSM (SCID) and the Mini-International Neuropsychiatric Interview (M.I.N.I). Eight studies, however, utilised self-report measures, such as the Generalised Anxiety Disorder-7 (GAD-7) scale to measure generalised anxiety, and the Short Health Anxiety Inventory (SHAI) to measure health anxiety.

### Prevalence of Anxiety by Country

There was no significant difference found (*p* = 0.28) in the prevalence estimates of anxiety in developed 30.4% (95% CI 27.0%—34.0%, *n* = 64), compared to developing 34.5% (95% CI 28.2%—41.4%, *n* = 17) countries.

### Prevalence of Anxiety by Sex, Mean Age Range and Year of Publication

**Fig. 5:**
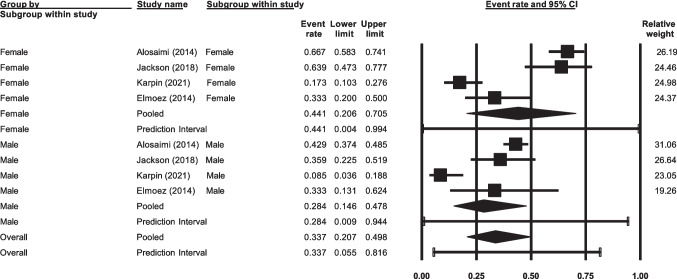
Prevalence of anxiety by sex

Only four studies reported prevalence of anxiety based on sex. In these studies, there was no significant difference found (*p* = 0.33) between females 44.1% (95% CI 20.6%—70.5%, *n* = 4) and males 28.4% (95% CI 14.6%—47.8%, *n* = 4; Fig. 5). Similarly, no significant difference was found in the prevalence of anxiety by patient’s mean age range (*p* = 0.55), with those in the 31–40 age group estimating 35.0% (95% CI 28.6%—42.1%, *n* = 22), those in the 41–50 age group estimating 33.4% (95% CI 28.3%—38.9%, *n* = 28), and those in the 51–60 age group estimating 29.3% (95% CI 22.3%—37.5%, *n* = 8). Only two studies mean age fell between 61–70, and so was not included in subgroup analysis.

Cumulative analysis was run by publication year to determine any variation over time (Appendix B), in conjunction with a subgroup analysis. No significant difference was found (*p* = 0.13).

## Discussion

Meta-analysis estimated an anxiety prevalence of 31.2% across all patients attending disease specific and general GI services – suggesting anxiety is a common experience amongst gastroenterology and hepatology patients. The prevalence is consistent with past literature [26, 27, 29, 30]. It is also consistent with anxiety estimates in other medical outpatient settings [18–21]. Notably, it is the highest value found in this review series. Whilst there is little research to suggest why this might be the case, a number of hypothesised factors may contribute to this, such as the nature of gastroenterology and hepatology symptoms being more chronic, long-term and unpredictable in nature, the stigmatisation and embarrassment associated with these conditions, and the difficulties patients have in gaining relief of their symptoms, thus having a significant impact on daily life [44–46]. Overall, anxiety appears to be a common experience across medical outpatients, and in particular gastroenterology and hepatology outpatients. Our point estimate for a general gastroenterology and hepatology clinic was somewhat lower, at 25.0%. Whilst this estimate may better reflect the general clinic setting, both values indicate anxiety is a common problem affecting gastroenterology and hepatology patients.

No significant difference was found across specific conditions, nor between gastroenterology and hepatology. However, we note prevalence estimates ranged (e.g., 25% in general gastroenterology and hepatology clinic settings, 31.5% in DGBI, 31.6% in IBD, 32.2% in hepatitis, and 43.4% in reflux). There are some variable results in literature regarding differences in anxiety rates between Crohn’s Disease and Ulcerative Colitis, however more commonly no significant difference is found between the two [29, 31], in keeping with present findings.

It is interesting that prevalence does not appear to vary across settings, despite variations in disease severity. This may suggest that anxiety rates are influenced more by the experience of living with chronic health conditions, rather than condition severity, or to currently undetermined immunological factors [47]. This may also be affected by factors such as heterogeneity influenced by demographic variables and sample sizes.

No significant difference was found across methods of assessment. This interestingly included no significant difference across different cutoffs in the HADS. This may be related to the variability across studies, or sample size. Variations in selected cut-offs for the HADS tool in primary research has been noted previously [18–21]. Research would benefit from studies using more standardised thresholds or reporting on each threshold.

Little research has used diagnostic interview methods to investigate prevalence. Further research using diagnostic interviews would be beneficial to compare across types of anxiety, and to obtain more accurate estimates, especially given self-report measures reportedly overestimate prevalence of anxiety [18, 20, 21].

A significant difference was found in prevalence across type of anxiety. Health anxiety was most common at 23.7%. This may relate to effects of these conditions on daily living, and the tendency to worry about symptoms and interoceptive sensations. For instance, IBS or IBD symptoms may cause a person to be alarmed by physiological sensations and their current and future impact on their health. Generalised anxiety followed at 14.5%, then specific phobias at 12.5%. Importantly, however, whilst the majority of these papers used diagnostic interviews, a handful included self-report measures, which again may have resulted in an overestimation of anxiety. In addition to this, the papers did not provide information on what specific phobias participants experienced. It would be helpful to further explore which specific phobias (e.g., injection phobia) are most common, and ensure gold standard approaches to assessment, using validated diagnostic interviews, are used. Nevertheless, these rates are notably higher than general population estimates; for example, a major study found a global point prevalence for experiencing *any* anxiety disorder of 4% [48].

No significant sex differences in anxiety rates were found, despite mean percentages appearing higher in females (44.1%) than males (28.4%). However, very limited literature was available to compare these rates. More studies are needed to assess whether this trend could be significant. Females have shown a higher anxiety prevalence than males in previous studies in our review series [19, 21], notably in respiratory and sleep medicine as well as in diabetic patients. However, no significant difference was found in cardiology and dermatology settings [18, 20]. Past literature has found sex differences across anxiety rates generally [49], and so this area may warrant further investigation, as does exploration of anxiety rates in trans and non-binary individuals.

Similarly, no significant difference was found in prevalence across human development index (HDI) level. Comparatively, significantly higher rates of anxiety have been found in developing nations in both the endocrinology and cardiology outpatient settings [19, 20]. However, there was more research performed in high (64) compared to low HDI countries (17). This is likely a reflection of resource availability for such research. If such barriers can be managed, further research in developing nations would be highly beneficial in better understanding anxiety globally.

No change in rates was found over years of publication, despite improving treatment. Like findings across condition type, this may suggest anxiety is driven more by the chronic health experience, or specifically for this speciality, the gut-brain axis. This aligns with literature that anxiety is common across chronic health condition patients [4]. However, the exact nature of this relationship cannot be fully understood from this data.

Experiencing anxiety disorders has an impact on wellbeing in several ways. In addition to direct impacts on wellbeing, increased anxiety levels within gastroenterology and hepatology disorders are associated with a greater symptom burden and higher utilisation of healthcare [50, 51]. This indicates that high prevalence rates are not only significant for mental health, but also in providing comprehensive physical medical care as well.

## Conclusions

Anxiety is a common condition amongst gastroenterology and hepatology outpatients, with an estimated prevalence of 31.2%. Anxiety was most prevalent amongst patients with reflux conditions, followed by hepatitis, then IBD. There was no significant variation across IBD conditions. Health anxiety was especially common at 23.7%. Prevalence estimates of many of the anxiety disorders in this population were substantially higher than estimates of anxiety overall in general populations, as well as other medical outpatient settings. Given the high prevalence of anxiety among gastroenterology and hepatology outpatients, and its significant impact on both physical and emotional wellbeing, it is therefore essential that healthcare providers recognise and address anxiety as a key component in their assessment and management of patients.

## Key References

- Romanazzo S, Mansueto G, Cosci F. Anxiety in the Medically Ill: A Systematic Review of the Literature. Front Psychiatry. 2022;13:873126. 10.3389/fpsyt.2022.873126.
  - ⚬ Comprehensive systematic review of the prevalence of anxiety symptoms and/or disorders for individuals with a medical illness. Highlights rates of anxiety across a range of gastrointestinal diseases as studied in the literature, including hepatitis, irritable bowel syndrome, inflammatory bowel disease, gastroesophageal reflux disease and chronic digestive system diseases.
- Barberio B, Zamani M, Black CJ, Savarino EV, Ford AC. Prevalence of symptoms of anxiety and depression in patients with inflammatory bowel disease: a systematic review and meta-analysis. The Lancet Gastroenterology & Hepatology. 2021;6(5):359–70. 10.1016/s2468-1253(21)00014-5.
  - ⚬ High quality systematic review and meta-analysis that investigates not only anxiety, but also depression, in patients with inflammatory bowel disease. Useful if interested in looking at other mental health disorders outside of anxiety to ensure appropriate screening.
- Storer B, Holden M, Kershaw KA, Braund TA, Chakouch C, Coleshill MJ, et al. The prevalence of anxiety in respiratory and sleep diseases: A systematic review and meta-analysis. Respiratory medicine. 2024:107677. 10.1016/j.rmed.2024.107677.
  - ⚬ The most recent publication in this review series, which focuses on the prevalence of anxiety in the respiratory and sleep medicine field. Also includes references to previous studies in the review series, including the dermatology, endocrinology, and cardiology settings. Useful if interested in comparing rates in the gastroenterology and hepatology field to other specialties.

## Supplementary Information

Below is the link to the electronic supplementary material.

Supplementary file1 (PDF 126 KB)

Supplementary file2 (PDF 128 KB)

Supplementary file3 (PDF 1079 KB)

## Data Availability

Details of included studies in this systematic review and meta-analysis are available in Table 1. Further information on the datasets used and/or analysed during the current study is available from the corresponding author upon reasonable request.
