# Supplementary material for: Global Prevalence of Anxiety in Gastroenterology and Hepatology Outpatients: A Systematic Review and Meta-Analysis
Source: Curr Gastroenterol Rep. 2025 Feb 27;27(1):17. doi: 10.1007/s11894-025-00963-x (PMC11868238; doi:10.1007/s11894-025-00963-x)
Supplement: Supplementary file 1 — Supplementary file1 (PDF 126 KB) [file 11894_2025_963_MOESM1_ESM.pdf]

Appendix A: Search Strategy and Criteria

Search strategy

This search was conducted as part of a review of anxiety across the five most common medical outpatient settings. Due to the substantial volume of results, the research team determined that each clinic would be reported separately, allowing for a more detailed analysis of results.

The research team began by identifying common medical outpatient specialties, measured by the number of specialists in each field in Australia. This data was taken from the Australian Institute of Health and Welfare's (AIHW) non-admitted patient care 2019-20 tables. (*Hospitals - Non-admitted patient care 2019-20 tables*, 2021) Papers that reported on general medicine outpatient settings, or across multiple types of clinic, were included if results were broken down by specialty or health conditions, such that relevant data could be extracted.

Studies that reported the prevalence of any form of anxiety (including somatic symptom disorder and somatoform disorders) were included for outpatient clinics or patient groups falling under; cardiology, gastroenterology and hepatology, endocrinology and diabetes, respiratory and sleep medicine, and dermatology. Due to the distinct nature of the presentation and patient experience in comparison to the other anxiety disorders discussed, body dysmorphic disorder was outside the scope of this review.

To enhance the search strategy, five common medical conditions in each specialty were included in the search, determined by a combination of AIHW data and clinical expertise.

A search strategy was developed in consultation with librarians at UNSW Sydney. Clinical keywords above were combined with relevant study types, anxiety terms, and outpatient search terms.

Studies which were relevant to the gastroenterology and hepatology setting were selected for this study. This included papers in this setting, as well as multi-specialty settings including gastroenterology and/or hepatology patient groups.

Inclusion criteria

- Study of patients in the medical outpatient setting, falling under cardiology, gastroenterology and hepatology, endocrinology and diabetes, respiratory and sleep medicine, or dermatology. General (internal) medicine settings were included, if a breakdown of data by condition (including relevant conditions) was available
- Study assesses prevalence of relevant anxiety symptoms or clinical disorders, using a validated assessment, including self-assessments, diagnostic interview/criteria, or report by a clinician
- Adult patients (≥18 years). Studies of patients ≥16 were included if the sample also included adult patients
- Any systematic reviews are included if, from reading the paper, the reviewer believes it is likely to yield papers that meet the inclusion criteria. These reviews' reference lists were then searched to identify relevant primary papers

Exclusion criteria

- Primary care, inpatients, emergency, or surgical settings
- Palliative care, end-of-life, or oncology settings

- Fertility clinics
- Papers of primarily psychiatric, addiction, or other mental health patients
- Selected patients are otherwise unrepresentative of the standard clinical cohort (e.g., pregnant patients, veterans)
- Study reports that data is available for less than 70% of selected eligible patients

Keywords

The search was conducted in PubMed, EMBASE, PsycInfo and Cochrane Library. The following search terms were used, requiring at least one positive match per column.

**Condition**	**Study type**	**Setting**	
anxiet*	Prevalence	Cardiology	Outpatient*
anxious	Incidence	Cardio* OR cardiac OR heart disease* OR heart clinic*	Ambulatory
Phobic disorder	Epidemiolog*	ischemic OR ischaemic	Clinic
Phobia	Population stud*	myocardial infarct*	
Panic	Cohort stud*	heart failure	
Post-traumatic stress OR Post traumatic stress	Cross-sectional stud*	cardiomyopath*	
OCD	Longitudinal stud*	arrythmia	
Obsessive	Observational stud*	Gastroenterology and hepatology	
Hypochondria*	Retrospective stud*	Gastroenterolog* OR bowel disease* OR stomach disease* OR liver disease* OR stoma disease* OR digestive disease* OR gastrointestinal	
Somatic symptom	Prospective stud*	inflammatory bowel	
Somati?ation	Descriptive stud*	irritable bowel	
Health anxiety	Frequency	reflux disease OR gastroesophageal reflux OR GERD	
Agoraphobi*		hepatitis	
Neurosis		chronic constipation	
Neuroses		Endocrinology	
Neurotic		Endocrinolog* OR hormon* disease* OR hormon* disorder*	
		thyroid disease OR hypothyroid OR hyperthyroid	
		polycystic ovar* OR PCOS	
		osteoporo*	
		diabet*	
		adrenal	
		Respiratory and sleep medicine	

		Pulmonary OR Respiratory OR Lung disease* OR sleep clinic	
		chronic obstructive OR COPD OR COAD	
		cystic fibrosis	
		asthma*	
		pneumonia	
		Dermatology	
		Dermatolog* OR skin disease*	
		acne	
		psoriasis	
		eczema	
		rosacea	
		Dermatitis	
		Dermatology	

PubMed

((anxiet*[Text Word]) OR (anxious[Text Word]) OR (Anxiety disorders[MeSH]) OR (Phobia[Text Word]) OR (Panic[Text Word]) OR (Post traumatic stress[Text Word]) OR (Post-traumatic stress[Text Word]) OR (OCD[Text Word]) OR (Obsessive[Text Word]) OR (Hypochondriasis[Text Word]) OR (Hypochondria*[Text Word]) OR (Somatic symptom[Text Word]) OR (Somati?ation[Text Word]) OR (Phobic disorder[Text Word]) OR (Agoraphobi*[Text Word]) OR (Neurosis[Text Word]) OR (Neuroses[Text Word]) OR (Neurotic[Text Word]))
**AND**<br>((Cohort stud*[Text Word]) OR (Cross-sectional stud*[Text Word]) OR (Epidemiolog*[Text Word]) OR (Incidence[Text Word]) OR (Longitudinal stud*[Text Word]) OR (Meta-anal*[Text Word]) OR (Observational stud*[Text Word]) OR (population stud*[Text Word]) OR (prevalence[Text Word]) OR (prospective stud*[Text Word]) OR (Retrospective stud*[Text Word]) OR (Systematic review[Text Word]) OR (Descriptive stud*[Text Word]) OR (frequency[Text Word]))
**AND**<br>((Cardio*[Text Word]) OR (cardiac[Text Word]) OR (heart disease*[Text Word]) OR (heart clinic*[Text Word]) OR (ischemic[Text Word]) OR (ischaemic[Text Word]) OR (myocardial infarct*[Text Word]) OR (heart failure[Text Word]) OR (cardiomyopath*[Text Word]) OR (arrythmia[Text Word]) OR (Gastroenterolog*[Text Word]) OR (bowel disease*[Text Word]) OR (stomach disease*[Text Word]) OR (liver disease*[Text Word]) OR (stoma disease*[Text Word]) OR (digestive disease*[Text Word]) OR (gastrointestinal[Text Word]) OR (inflammatory bowel[Text Word]) OR (irritable bowel[Text Word]) OR (reflux disease[Text Word]) OR (gastroesophageal reflux[Text Word]) OR (GERD[Text Word]) OR (hepatitis[Text Word]) OR (chronic constipation[Text Word]) OR (Endocrinolog*[Text Word]) OR (hormon* disease*[Text Word]) OR (hormon* disorder*[Text Word]) OR (thyroid disease[Text Word]) OR (hypothyroid[Text Word]) OR (hyperthyroid[Text Word]) OR (polycystic ovar*[Text Word]) OR (PCOS[Text Word]) OR (osteoporo*[Text Word]) OR (diabet*[Text Word]) OR (adrenal[Text Word]) OR (Pulmonary[Text Word]) OR (Respiratory[Text Word]) OR (Lung disease*[Text Word]) OR (sleep clinic[Text Word]) OR (chronic obstructive[Text Word]) OR (COPD[Text Word]) OR (COAD[Text Word]) OR (cystic fibrosis[Text Word]) OR (asthma*[Text Word]) OR (pneumonia[Text Word]) OR (Dermatolog*[Text Word]) OR (skin disease*[Text Word]) OR (acne[Text Word]) OR (psoriasis[Text Word]) OR (eczema[Text Word]) OR (rosacea[Text Word]) OR (Dermatitis[Text Word]))
**AND**<br>((Outpatients[MeSH]) OR (Outpatient Clinics, Hospital[MeSH]) OR (Outpatient*[Text word]) OR (Ambulatory care[MeSH]) OR (Ambulatory Care Facilities[MeSH]) OR (Ambulatory[text word]) OR (Outpatient Clinics, Hospital[MeSH]) OR (Clinic[Text Word]))

Filters: adult, human papers

PsycINFO

(AB,TI,SU(anxiet*) OR AB,TI,SU(anxious) OR AB,TI,SU(Phobia) OR AB,TI,SU(Panic) OR AB,TI,SU(Post traumatic stress) OR AB,TI,SU(Post-traumatic stress) OR AB,TI,SU(PTSD) OR AB,TI,SU(OCD) OR AB,TI,SU(Obsessive) OR AB,TI,SU(Hypochondriasis) OR AB,TI,SU(Hypochondria*) OR AB,TI,SU(Somatic symptom) OR AB,TI,SU(Somati?ation) OR AB,TI,SU(Phobic disorder) OR AB,TI,SU(Agoraphobi*) OR AB,TI,SU(Neurosis) OR AB,TI,SU(Neuroses) OR AB,TI,SU(Neurotic))
**AND**<br>(AB,TI,SU(Cohort stud*) OR AB,TI,SU(Cross-sectional stud*) OR AB,TI,SU(Epidemiolog*) OR AB,TI,SU(Incidence) OR AB,TI,SU(Longitudinal stud*) OR AB,TI,SU(Meta-anal*) OR AB,TI,SU(Observational stud*) OR AB,TI,SU(population stud*) OR AB,TI,SU(prevalence) OR AB,TI,SU(prospective stud*) OR AB,TI,SU(Retrospective stud*) OR AB,TI,SU(Systematic review) OR AB,TI,SU(Descriptive stud*) AB,TI,SU(Frequency))
**AND**<br>(AB,TI,SU(Cardio*) OR AB,TI,SU(cardiac) OR AB,TI,SU(heart disease*) OR AB,TI,SU(heart clinic*) OR AB,TI,SU(ischemic) OR AB,TI,SU(ischaemic) OR AB,TI,SU(myocardial infarct*) OR AB,TI,SU(heart failure) OR AB,TI,SU(cardiomyopath*) OR AB,TI,SU(arrythmia) OR AB,TI,SU(Gastroenterolog*) OR AB,TI,SU(bowel disease*) OR AB,TI,SU(stomach disease*) OR AB,TI,SU(liver disease*) OR AB,TI,SU(stoma disease*) OR AB,TI,SU(digestive disease*) OR AB,TI,SU(gastrointestinal) OR AB,TI,SU(inflammatory bowel) OR AB,TI,SU(irritable bowel) OR AB,TI,SU(reflux disease) OR AB,TI,SU(gastroesophageal reflux) OR AB,TI,SU(GERD) OR AB,TI,SU(hepatitis) OR AB,TI,SU(chronic constipation) OR AB,TI,SU(Endocrinolog*) OR AB,TI,SU(hormon* disease*) OR AB,TI,SU(hormon* disorder*) OR AB,TI,SU(thyroid disease) OR AB,TI,SU(hypothyroid) OR AB,TI,SU(hyperthyroid) OR AB,TI,SU(polycystic ovar*) OR AB,TI,SU(PCOS) OR AB,TI,SU(osteoporo*) OR AB,TI,SU(diabet*) OR AB,TI,SU(adrenal) OR AB,TI,SU(Pulmonary) OR AB,TI,SU(Respiratory) OR AB,TI,SU(Lung disease*) OR AB,TI,SU(sleep clinic) OR AB,TI,SU(chronic obstructive) OR AB,TI,SU(COPD) OR AB,TI,SU(COAD) OR AB,TI,SU(cystic fibrosis) OR AB,TI,SU(asthma*) OR AB,TI,SU(pneumonia) OR AB,TI,SU(Dermatolog*) OR AB,TI,SU(skin disease*) OR AB,TI,SU(acne) OR AB,TI,SU(psoriasis) OR AB,TI,SU(eczema) OR AB,TI,SU(rosacea) OR AB,TI,SU(Dermatitis))
**AND**<br>(AB,TI,SU(Outpatients) OR AB,TI,SU(Outpatient Clinics, Hospital) OR AB,TI,SU(Outpatient*) OR AB,TI,SU(Ambulatory care) OR AB,TI,SU(Ambulatory Care Facilities) OR AB,TI,SU(Ambulatory) OR AB,TI,SU(Outpatient Clinics, Hospital) OR AB,TI,SU(Clinic))

Filters: adult papers

Cochrane

anxiet* OR anxious OR Phobia OR Panic OR "Post traumatic stress" OR "Post-traumatic stress" OR PTSD OR OCD OR Obsessive OR Hypochondria* OR “Somatic symptom” OR Somati?ation OR “Phobic disorder” OR agoraphobi* OR Neurosis OR Neuroses OR Neurotic
**AND**<br>(cohort or cross-sectional or longitudinal or observational or population or prospective or retrospective or descriptive) NEXT stud* or epidemiolog* or incidence or “meta-analysis” or “meta-analyses” or prevalence or “systematic review” or frequency
**AND**<br>(Cardio*) OR (cardiac) OR (heart NEXT disease*) OR (heart NEXT clinic*) OR (ischemic) OR (ischaemic) OR (myocardial NEXT infarct*) OR ("heart failure") OR (cardiomyopath*) OR (arrythmia) OR (Gastroenterolog*) OR (bowel NEXT disease*) OR (stomach NEXT disease*) OR (liver NEXT disease*) OR (stoma NEXT disease*) OR (digestive NEXT disease*) OR (gastrointestinal) OR ("inflammatory bowel") OR ("irritable bowel") OR ("reflux disease") OR ("gastroesophageal reflux") OR (GERD) OR (hepatitis) OR ("chronic constipation") OR (Endocrinolog*) OR (hormon* NEXT disease*) OR (hormon* NEXT disorder*) OR ("thyroid disease") OR (hypothyroid) OR (hyperthyroid) OR (polycystic NEXT ovar*) OR (PCOS) OR (osteoporo*) OR (diabet*) OR (adrenal) OR (Pulmonary) OR (Respiratory) OR (Lung NEXT disease*) OR ("sleep clinic") OR ("chronic obstructive") OR (COPD) OR (COAD) OR ("cystic fibrosis") OR (asthma*) OR (pneumonia) OR (Dermatolog*) OR (skin NEXT disease*) OR (acne) OR (psoriasis) OR (eczema) OR (rosacea) OR (Dermatitis)
**AND**<br>Outpatient* OR Ambulatory OR Clinic

Filters: none

EMBASE

exp anxiety disorder/ or exp anxiety/ or anxiet*.mp. or anxious.mp. or exp phobia/ or phobi*.mp. or exp panic/ or panic.mp. or Post traumatic stress.mp. or PTSD.mp. or exp posttraumatic stress disorder/ or OCD.mp. or exp obsession/ or exp obsessive compulsive disorder/ or obsessive.mp. or exp hypochondriasis/ or hypochondria*.mp. OR somatic symptom.mp. or somati?ation.mp. or exp somatization/ or exp agoraphobia/ or agoraphobi*.mp. or exp neurosis/ or neuros?s.mp. or neurotic.mp.
**AND**<br>Cohort stud*.mp. or Cross-sectional stud*.mp. or Epidemiolog*.mp. or Incidence.mp. or Longitudinal stud*.mp. or Meta-anal*.mp. or Observational stud*.mp. or population stud*.mp. or prevalence.mp. or prospective stud*.mp. or Retrospective stud*.mp. or Systematic review.mp. or frequency.mp. or descriptive stud*.mp.
**AND**<br>Cardio*.mp. OR cardiac.mp. OR heart disease*.mp. OR heart clinic*.mp. OR ischemic.mp. OR ischaemic.mp. OR myocardial infarct*.mp. OR heart failure.mp. OR cardiomyopath*.mp. OR arrythmia.mp. OR Gastroenterolog*.mp. OR bowel disease*.mp. OR stomach disease*.mp. OR liver disease*.mp. OR stoma disease*.mp. OR digestive disease*.mp. OR gastrointestinal.mp. OR inflammatory bowel.mp. OR irritable bowel.mp. OR reflux disease.mp. OR gastroesophageal reflux.mp. OR GERD.mp. OR hepatitis.mp. OR chronic constipation.mp. OR Endocrinolog*.mp. OR hormon* disease*.mp. OR hormon* disorder*.mp. OR thyroid disease.mp. OR hypothyroid.mp. OR hyperthyroid.mp. OR polycystic ovar*.mp. OR PCOS.mp. OR osteoporo*.mp. OR diabet*.mp. OR adrenal.mp. OR Pulmonary.mp. OR Respiratory.mp. OR Lung disease*.mp. OR sleep clinic.mp. OR chronic obstructive.mp. OR COPD.mp. OR COAD.mp. OR cystic fibrosis.mp. OR asthma*.mp. OR pneumonia.mp. OR Dermatolog*.mp. OR skin disease*.mp. OR acne.mp. OR psoriasis.mp. OR eczema.mp. OR rosacea.mp. OR Dermatitis.mp.
**AND**<br>exp outpatient care/ or Outpatient*.mp. or exp outpatient/ or exp outpatient department/ or exp ambulatory care/ or ambulatory.mp. or clinic.mp.

Filters: adult papers

References

Hospitals - Non-admitted patient care 2019-20 tables. (2021). Retrieved from: https://www.aihw.gov.au/getmedia/70b1f972-03be-4de8-be71-92a80dfea373/Non-admitted-patient-care-2019-20.xls.aspx
