# Supplementary figures and images for: Global Prevalence of Anxiety in Gastroenterology and Hepatology Outpatients: A Systematic Review and Meta-Analysis
Source: Curr Gastroenterol Rep. 2025 Feb 27;27(1):17. doi: 10.1007/s11894-025-00963-x (PMC11868238; doi:10.1007/s11894-025-00963-x)

Funnel Plot of Precision by Logit event rate

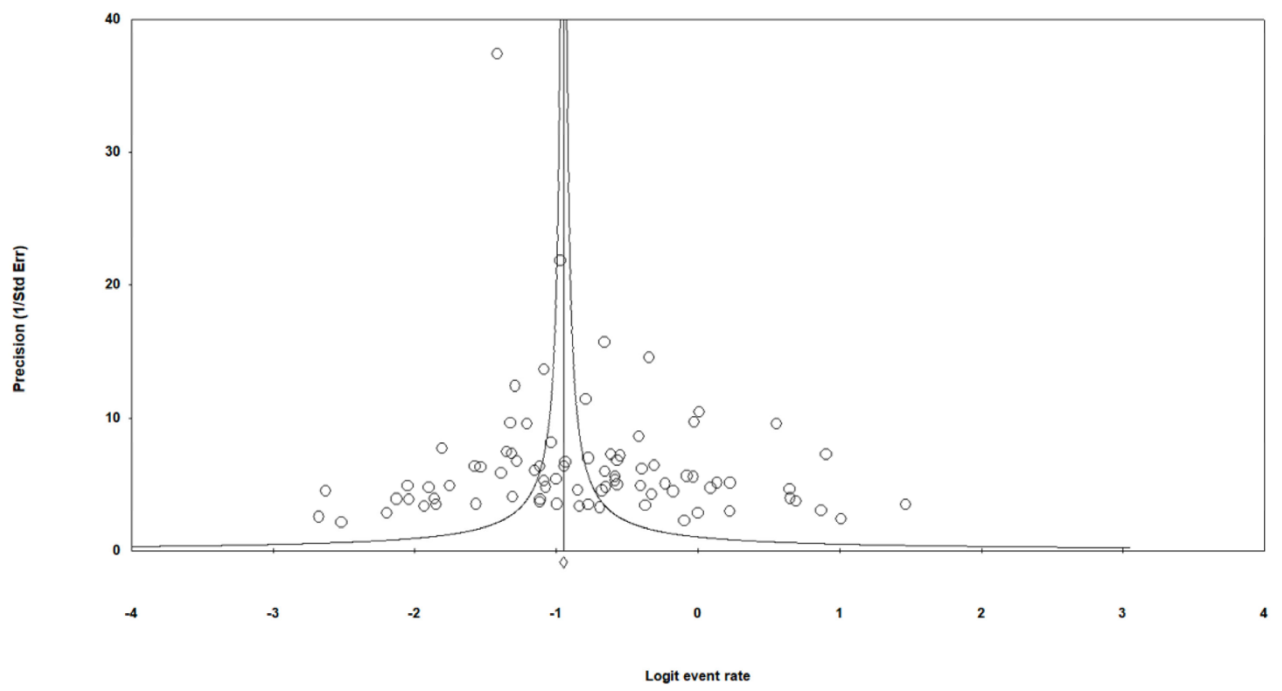

Supplement: Supplementary file 2 — Supplementary file2 (PDF 128 KB) [file 11894_2025_963_MOESM2_ESM.pdf]

# Meta Analysis

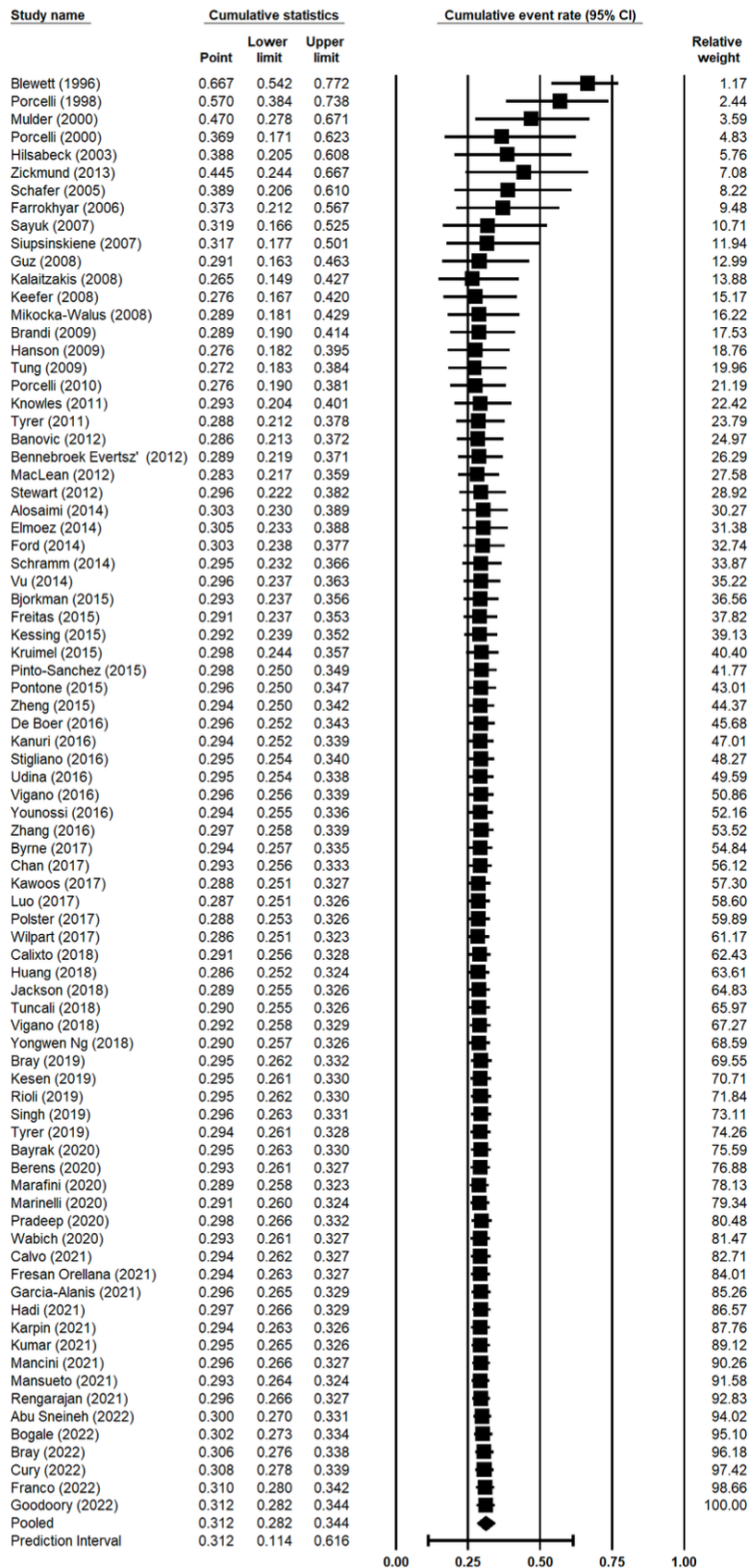

Supplement: Supplementary file 3 — Supplementary file3 (PDF 1079 KB) [file 11894_2025_963_MOESM3_ESM.pdf]
